# A Network Model of Goals Boosts Convergent Creativity Performance

**DOI:** 10.3389/fpsyg.2018.01910

**Published:** 2018-10-29

**Authors:** Franki Y. H. Kung, Abigail A. Scholer

**Affiliations:** ^1^Department of Psychological Sciences, Purdue University, West Lafayette, IN, United States; ^2^Department of Psychology, University of Waterloo, Waterloo, ON, Canada

**Keywords:** goal models, goal structure, network model, goal regulation, creativity, multiple goals

## Abstract

To increase employee creativity is critical for organizational success, and yet we still know very little about what organizational contexts promote creative performance. Our research proposes that goal regulation in the workplace may have consequences for creativity. While there is an increasing trend for organizations and workers to visualize the structure of their goals (e.g., management hierarchy, concept-map, flowchart), prior research suggests the visualization approaches differ as one of the three types: hierarchical, network, and sequential models. Because a network model (vs. hierarchical and sequential models) highlights multiple connections between goals and reveals unobvious connections between them, we hypothesized that the use of a network goal model might increase people’s ability to integrate seemingly unrelated ideas, even on subsequent unrelated tasks, leading to higher (convergent) creative performance. To test the hypothesis, we conducted an experiment in 2017 manipulating participants’ goal models (hierarchical, network, sequential; *N* = 191, median age = 19) and measured their creativity. Results suggest that those in the network model condition performed better in the kind of creativity task that requires meaningful integration of unrelated ideas (i.e., convergent creativity); in contrast, there was no difference between goal model conditions on divergent creative performance. These findings thus illuminate how goal models may influence creativity, providing new insights into situational inductions that can boost creative performance. Theoretical and practical implications, limitations, and future directions of the work are discussed.

## Introduction

Increasing employee creativity is important to organizational effectiveness (Runco, 2004a; Anderson et al., 2014), and organizations are often looking for ways to boost employee creativity (Hennessey and Amabile, 2010). Research has traditionally emphasized creativity as an outcome of relatively stable dispositional traits (vs. states; Barron and Harrington, 1981; Woodman et al., 1993; Runco, 2004b). We know relatively little about situational factors that facilitate creativity (c.f. Kray et al., 2006; Mueller et al., 2014), especially in terms of strategies that are both effective and efficient (e.g., Scott et al., 2004). The current research examines how people’s mindsets about how their goals are generally related (i.e., goal structure) may affect creativity on subsequent, unrelated tasks.

Our approach proposes that one factor influencing employee creativity may arise as a (often unintended) consequence of goal regulation in the workplace. Specifically, we argue that the structures people use to organize their goals – goal models – may affect subsequent creative performance. Prior work suggests that goal models typically emerge as one of three types: hierarchical, network, or sequential models (Table 1; Kung and Scholer, 2016). Each model emphasizes a different aspect of goal relations (importance, association, timing) with significant implications for self-regulation (Kung, 2018). These variations in lay theories of goal structure are also consistent with distinct principles emphasized in business and management approaches to goal visualization. Indeed, a surging trend for organizations and workers to visualize the structure of their goals (e.g., business/management hierarchy, concept-map, flowchart; Dettmer, 2007; Asana, 2018; Mindmeister, 2018b) suggests the relevance of examining how such processes may have spillover effects on creative performance.

**Table 1:** A goal model framework.

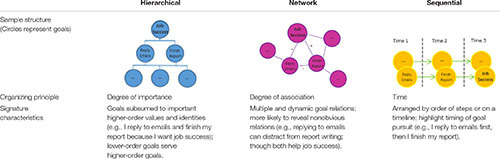

In particular, we propose that the adoption of network goal models (relative to hierarchical and network models) is likely to increase (convergent) creative performance. A network model highlights multiple connections between goals and reveals unobvious connections between them; therefore, the use of a network goal model might increase people’s ability to integrate seemingly unrelated ideas, even on subsequent tasks, leading to higher (convergent) creative performance. The results of the current experiment thus shed light on how goal models may influence creativity, offering new insights into situational inductions that can boost creative performance.

### Emerging Goal Models in Organizations

**FIGURE 1:**
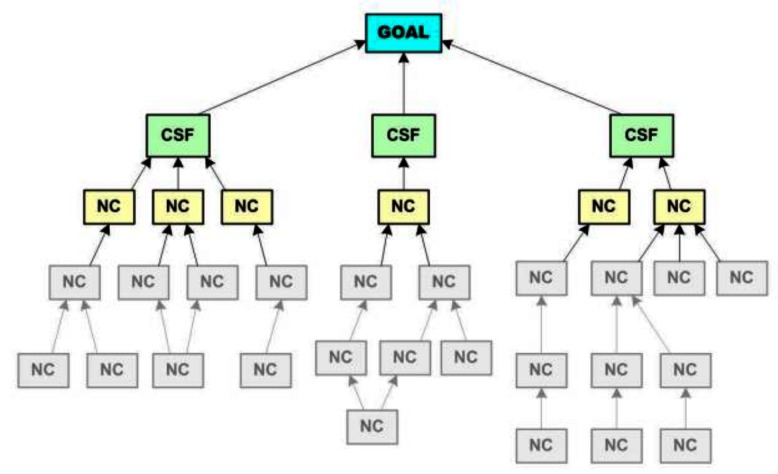
A hierarchical company goal tree (Dettmer, 2007). CSF, critical success factor; NC, necessary condition of the goal.

Many organizations and workers structure their goals in some ways to facilitate the understanding of personal and shared goals, and these structures vary in the underlying organizing principles. Work on lay theories of goal models differentiates these principles into three major categories – hierarchical, network, or sequential models (Kung and Scholer, 2016; Kung, 2018; Table 1). *Hierarchical models* emphasize the principle of importance as the key feature of goal relations – identifying the value behind the pursuit of the goal (e.g., vision and mission) and focusing on ways to get there (McKeown, 2014). In hierarchical models, concrete actions are subsumed into more abstract values (Powers, 1973; Carver and Scheier, 1982; Vallacher and Wegner, 1989). Warren Buffet, a well-known CEO and investor, once shared the (hierarchical) principle he used to organize his goals, ranking goals from the most to least important and focusing on achieving the most important goals (Schroeder, 2008). In business, these are sometimes called “strategic plans,” “business reference models,” “goal trees,” or “intermediate goal models” (Figure 1). It is not uncommon to see companies use a hierarchy to structure their missions (Dettmer, 2007; Hierarchystructure, 2018) and workers also use hierarchical models to organize their tasks (Bateman et al., 2002; Bagozzi et al., 2003; Duckworth, 2016).

**FIGURE 2:**
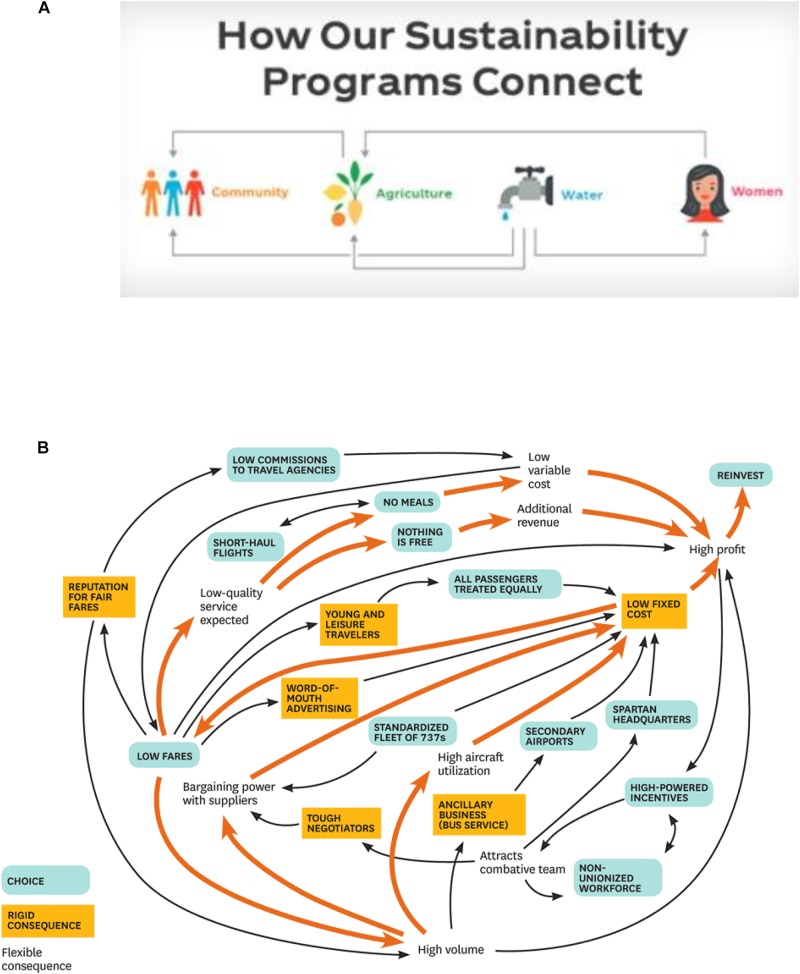
**(A)** A network model of sustainability goals (The Coca-Cola Company, 2018). Picture copyrighted by the Coca-Cola Company, https://www.coca-colacompany.com/sustainability. **(B)** A network business model of Ryanair (Casadesus-Masanell and Ricart, 2011).

Another type of goal model, *network models*, are those that emphasize the principle of association. Compared to hierarchical models, network models have a more horizontal structure, highlighting multiple possible relations among goals and revealing both positive and negative relations among them (Collins and Loftus, 1975; Higgins et al., 1985). In business, people sometimes call these network models “concept/mind maps” or “business models” (also called an “*N*-squared diagram” in systems-engineering language). Project management software that helps teams to create network model-like visualizations for their work tasks, such as “Mindmeister”^1^, is increasingly popular and now used in global organizations like CNN and Oracle (Mindmeister, 2018b). These visualizations are supposed to increase worker productivity because they highlight the contingency of work within a person or team, as well as how tasks within a person or team both facilitate and hinder each other (Mindmeister, 2018a). At the company level, organizations can communicate their business operations visually based on how functions of their task connect. The Coca-Cola Company (Figure 2A) visualizes how their sustainability goals are connected (e.g., water resource can both directly and indirectly – through improving lives of women – facilitates local agriculture). Simiarly, Ryanair (Figure 2B) illustrates how business decisions are intertwined (e.g., a decision can be a trigger and an outcome at the same time), posing constraints on one another. These are examples of the use of a network model.

**FIGURE 3:**
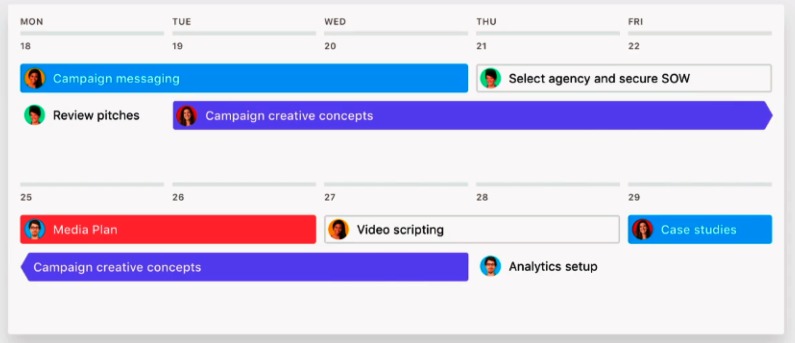
A timeline of worker goals (Asana, 2018). Picture copyrighted by Asana, Inc., https://asana.com/product.

The last type of goal model, *sequential models*, emphasize the principle of time – identifying the stages and timing of achieving one’s goals. A sequential model usually arranges goals in chronological steps so that the timing for pursuing a specific goal is clear (Gollwitzer, 1990; Heckhausen et al., 2010). David Allen, a management consultant famous for his productivity method known as “getting things done,” popularized a method similar to a sequential model view of goals: the rule of productivity is to recognize how long a task takes and decide the ideal timing to do it (Allen, 2015). In business, a sequential model of goals can be as common as the informal use of a personal calendar; it may also function more formally as an organizational “run charts,” “flow process charts,” or “business process models.” Project management software like “Airtable”^2^, “Asana”^3^, “Targetprocess”^4^, and “Trello”^5^ can help workers to create personal and team calendars to visualize the current state of goal progress (Figure 3). These programs are now used by large organizations such as Google (Trello, 2018), Deloitte, and NASA (Asana, 2018).

Prior research reveals that these three types of goal models are the most common goal structures that people spontaneously generate and endorse (Kung and Scholer, 2016). This is not to suggest that these three goal models are exhaustive, nor that there can also be instances of integrated models. However, people’s lay theories about the nature of relations among their goals frequently emphasize one of these principles in particular. Furthermore, even though these models are not veridical reflections of how goals are organized in the brain, we know from classic psychology theories that subjective perceptions are powerful and can have long-reaching impact of thinking and behavior (e.g., Heider, 1958; Kelley, 1973; Kahneman and Tversky, 1979). For this reason, goal models may have not only direct implications for how people regulate their goals, but also have indirect implications for how people think and behave in general. As such, goal models may affect how people approach problems and find creative solutions. In particular, based on cognitive theories of creativity, we propose that network models (vs. the other two) may have implications for creative performance.

### Goal Models and Creativity

Creativity usually happens in two related but distinct forms: divergent and convergent creativity (see Eysenck, 2003). Divergent creativity is the ability to generate multiple distinct ideas from a single source (e.g., coming up with many uses of a brick), whereas convergent creativity refers to the ability to combine seemingly unrelated ideas into a meaningful idea (e.g., combining the use of bricks and concrete to build a house). Whereas both types of creativity are essential, convergent creativity involves the complex skill of both generating and synergizing to combine seemingly unrelated ideas into a meaningful entity (Mednick, 1962; Cropley, 2006). Convergent creativity predicts critical indicators of job performance such as knowledge integration and problem-solving, and therefore is particularly valued at work (Cropley, 2006).

However, research suggests that convergent creativity can be hard to come by because individuals are typically biased to ignore interconnections between ideas (Tadmor et al., 2012a,b; Mellers et al., 2014). For instance, workers tend to falsely place more emphasis on their initial ideas, and therefore, they fail to converge initial ideas with subsequent insights for a more creative product (e.g., Berg, 2014). In addition, people tend to have exaggerated perceptions of how different opposing ideas are (Thompson, 1990). When they see ideas as vastly different, they often lack the motivation or insight to integrate these seemingly unrelated ideas (Tierney and Farmer, 2002; Tadmor et al., 2012a,b). As a result of these biases, many people miss out on creative integrative solutions. In order to overcome these biases and attain better creative performance, people need to be open to the possibility of multiple relations among ideas, with the capacity to flexibly draw interconnections between ideas.

The use of goal models may have the capacity to influence the way people approach the relations between ideas, and thus, creative performance. Ample research suggests that cognitive properties, such as thinking styles and motivation, can extend from one domain to another (see Baumann and Kuhl, 2005; Förster et al., 2008; Trope and Liberman, 2010). For instance, studies showed that properties in the physical domain can spill over to the social domains: perceptions of spatial distance increased feelings of emotional distance (Williams and Bargh, 2008) and attention to global features in pictures increased the use of social stereotypes to evaluate a target person (McCrea et al., 2012). Likewise, the properties of the way people visualize the relations among their goals (as a function of their goal models) may extend to affect mental processes involved in the domain of creative problem-solving in general.

In particular, the use of a network goal model may provide an avenue for convergent creativity to arise. As discussed earlier, a network model organizes goals by association. It tends to draw people’s attention to interconnections between ideas, revealing nonobvious connections between them (Kung and Scholer, 2016). This nature of network goal model can be conducive to convergent creativity for two reasons. First, connecting goals by association, network models have fewer restrictions on both how and why goals are connected (Collins and Loftus, 1975). Goals can be connected in a network simply by semantic or domain relevance. Because a network structure is a flat structure – allowing ideas to be on a relatively equal playing field – it is likely to liberate people from pre-existing assumptions (e.g., some ideas are more important than the others; Berg, 2014) and incubate creative integration of different ideas. Second, network models highlight interconnected goal relations. Supporting this notion, past research revealed that people who spontaneously used a network model to organize their goals discovered more relations among their goals (Kung, 2018). The awareness of interconnections among their goals, when transferred to the domain of creative problem-solving, may also allow people to more easily see connections between seemingly unrelated ideas. All these factors would suggest that the use of a network model should increase people’s ability to achieve higher convergent creativity.

### An Empirical Test

The current research tested the effect of goal models on creative performance. We developed a goal model-induction exercise (based on mind-mapping techniques; Trochim, 1989) to manipulate goal models and then measured subsequent creative performance. To examine the boundary conditions of the network model effect, we included measures of both convergent and divergent creativity. As the salience of interconnections among diverse ideas should be more closely related to convergent than divergent thinking (Cropley, 2006), we examined if the network mindset would have a stronger effect on convergent (vs. divergent) creative performance.

## Materials and Methods

### Power, Participants, and Design

This experiment had a between-subjects design (Condition: network, hierarchical, sequential models). We launched the study recruitment via the University of Waterloo Psychology Participant Pool in 2017 and undergraduate participants signed up to complete a lab study for one course-credit. In two semesters, 239 undergraduates participated in the study. Among them, 36 had previously completed the divergent creativity task and were ineligible for analysis (Steffens et al., 2016), and an additional 12 had missing data on the key measures. Excluding these participants resulted in a final sample of 191 for analysis (72% female; Median age = 19; 48% Asian, 35% White, 13% Others, and 4% Black). A sensitivity power analysis showed that this sample size gave us 80% power to detect a minimum small-to-medium-effect-size difference ($\eta_{p}^{2}$ = 0.05 or *d* = 0.44) between network versus the other two conditions, at the 0.05 alpha error probability (two-tailed; Faul et al., 2009).

Participants came to the lab and were randomly assigned to complete one of the three conditions of the goal model manipulation task. Afterward, they completed a convergent creativity task and a divergent creativity task on the computer that were unrelated to the goal model induction. The order of the two creativity tasks was randomized by the computer and did not affect the pattern of the results. Finally, participants completed a battery of exploratory measures, which included manipulation check questions about the goal model manipulation task.

### Goal Model Manipulation

**FIGURE 4:**
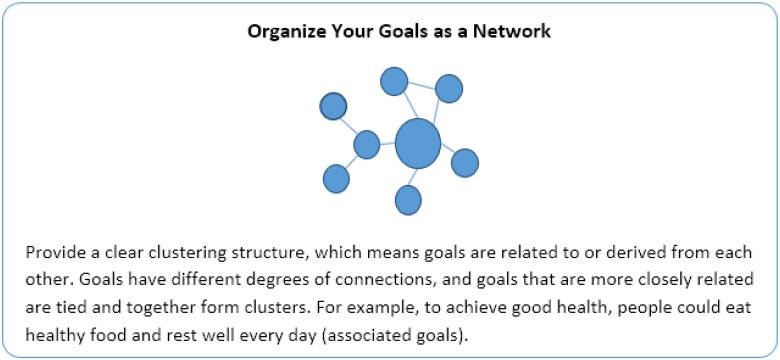
Goal model manipulation task descriptions: network model condition.

Participants came to the lab and received a paper-and-pencil booklet that asked them to create a visualization of what they did at school to achieve their goal of university success. The task takes only about 10 min and is similar to a mind-mapping exercise (Trochim, 1989). Participants were randomly assigned to one of the three conditions: network, hierarchical, or sequential model. As illustrated in Figure 4, they received a prototype figure of one of three goal models (with a brief explanation of the structure) and were told to follow the structure of the figure to create their goal model (see full materials in Supplementary Material).

**FIGURE 5:**
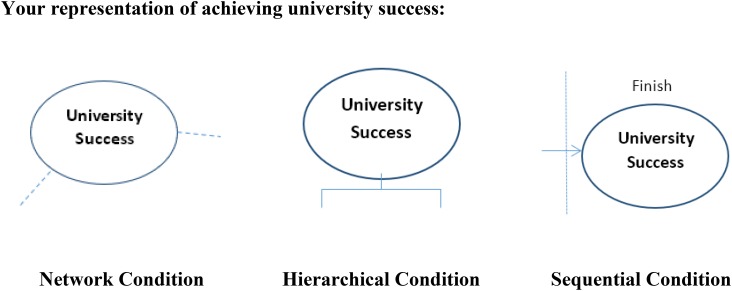
The unfinished diagrams for participants to complete in the goal model manipulation task.

To make it more intuitive for participants to follow the instructions, the task provided an unfinished diagram – as seen in Figure 5 – where the focal goal was indicated according to the prototype. Participants completed the diagram and drew as many goals as they wanted. In essence, this manipulation kept the focal goal across conditions constant, while altering the structure participants used to organize their focal goal in relation to other idiosyncratic goals (see Supplementary Material for sample diagrams from participants).^6^

#### Goal Importance

To explore whether there were differences in single goal properties across goal models (that might influence the hypothesized result), we included an item measuring goal importance following the goal manipulation task. Goal importance captures many vital goal content properties, such as goal commitment (Talevich et al., 2014) and abstractness (Vallacher and Wegner, 1987). For each of the goals on the goal map, participants answered the question of “How important is each goal to you at this point in your life?” from 1 (*Not at all important*) to 11 (*Very important*).

##### Manipulation check

As a manipulation check, we included items at the end of the experiment to measure the degree to which participants followed certain goal organizing principles when creating their goal model. Participants responded to each item on a scale from 1 = *Strongly disagree* to 7 = *Strongly agree*. The items measured the use of the principle of goal importance (4 items, α = 0.91; e.g., “I classified my goals by order of importance.”), the principle of goal interconnections (four items, α = 0.74; e.g., “I paid a lot of attention to the ways that goals were related to each other.”), and the principle of time (four items, α = 0.91; e.g., “I organized my goals in chronological orders”; see full scale in Appendix A).^7^

### Convergent Creative Thinking

Convergent creativity was measured in a creative story-rewriting task (Leung and Chiu, 2010). Participants were given a short summary of the story of Snow White. They were told to use their wildest imagination to rewrite the story, but the story needed to be based on the original fairy tale (see the task in Supplementary Material). In doing so, this task challenged participants’ ability to connect seemingly unrelated ideas, however diverse they are, to form a new and coherent story in their own version. Hence, the outcome of the task can be operationalized as convergent creative thinking.

To evaluate participants’ performance on the task, we recruited and trained four coders who were blind to the hypothesis and to participant condition to evaluate each participant’s story independently and in random order. For each story, the coders rated creative performance on a seven-point scale, from 1 = *Not at all* to 7 = *Extremely creative* (Leung and Chiu, 2010). The reliability among the coders was high (α = 0.85), so we averaged their ratings to form a convergent creativity index for each participant.

### Divergent Creative Thinking

Divergent creativity was measured with the standard unusual uses task (Guilford, 1950; Torrance, 1974). In this task, participants were given 2 min to generate as many creative uses of a brick as possible (see Supplementary Material). This task challenged participants’ ability to expand the one central idea (i.e., the brick) to as many and as diverse ideas as possible, without any constraint on the interconnections and coherence between these ideas. Therefore, the outcome of the task can be operationalized as divergent creative thinking. To evaluate participants’ performance on the task, we recruited and trained three coders (different from the coders employed for coding convergent creative thinking) who were blind to the hypothesis and to participant condition to evaluate each use independently and in random order.

A participant’s performance in the unusual uses task was the combination of three sub-scores: fluency, flexibility, and originality (Torrance, 1974; Kurtzberg, 1998; Tadmor et al., 2009). The fluency sub-score was the number of ideas each participant generated. The flexibility sub-score was the number of semantic categories a participant used out of a list of 27 categories (e.g., using a brick as a weapon, as a doorstep). The list was adopted from a pre-existing list of 19 categories (Markman et al., 2007), with an addition of eight categories generated in consensus by the coders to fit all participants’ uses (e.g., smoothing tool, extinguishing tool). The interrater reliability, bbb = 0.82, was substantial (Landis and Koch, 1977; Fleiss et al., 2003). The coders discussed the discrepancy and agreed on the final category of each use participants generated. Finally, the originality sub-score was measured by coders’ subjective evaluation of how novel the use was on a scale from 1 = *Not at all* to 7 = *Extremely creative* (α = 0.79). The originality score of a participant was the average score across all the coders and the uses participants generated. These three creativity scores were highly consistent (α = 0.85). Hence, the average of the three standardized sub-scores for each participant formed the index of divergent creative thinking.

## Results

### Manipulation Check

To test the effectiveness of the goal model manipulation, we examined ratings of goal organizing principles (within-subjects: importance, interconnection, and time) as a function of the goal model condition (between-subjects: hierarchical, network, and sequential). Because of the mixed design, we conducted a mixed-model ANOVA. Results showed a significant interaction, *F*(2,668) = 88.80, *p* < 0.001, $\eta_{p}^{2}$ = 0.21, suggesting that participants’ goal organizing principles differed depending on their goal model condition.

To unpack the result, we created dummy variables to contrast each goal model with the other two (e.g., Hierarchical Model: hierarchical = 1, network = 0, sequential = 0). These variables allow the significant test of the difference between the target goal model and the two other models. Results of the *t*-tests are reported in Table 2.

**Table 2:** Independent *t*-tests: manipulation check analyses, goal model condition predicting goal organizing principles.

DV	Predictor	*M*	*SD*	*B*	*SE*	*t*	*p*	95% CI	$\eta_{p}^{2}$
Importance	1. Hierarchical	4.67	1.37	0.82***	0.24	3.42	0.001	[0.35, 1.29]	0.06
	2. Network	3.74	1.72	-0.58*	0.25	-2.36	0.019	[-1.07, -0.10]	0.03
	3. Sequential	3.96	1.62	-0.26	0.25	-1.05	0.296	[-0.75, 0.23]	0.01
Interconnection	1. Hierarchical	4.62	1.21	-0.18	0.18	-1.03	0.307	[-0.53, 0.17]	0.01
	2. Network	4.92	1.13	0.26	0.18	1.43	0.154	[-0.10, 0.61]	0.01
	3. Sequential	4.69	1.17	-0.07	0.18	-0.38	0.702	[-0.43, 0.29]	<0.01
Time	1. Hierarchical	3.57	1.47	-0.71**	0.26	-2.72	0.007	[-1.23, -0.20]	0.04
	2. Network	3.42	1.41	-0.90***	0.26	-3.44	0.001	[-1.42, -0.39]	0.06
	3. Sequential	5.12	1.82	1.62***	0.24	6.71	<0.001	[1.15, 2.10]	0.19

N = 191: 66 hierarchical (35%), 62 network (32%), and 63 sequential (33%). Predictors are dummy-coded (e.g., Hierarchical Model: hierarchical = 1, network = 0, sequential = 0). For each dependent variable, individual tests are numbered separately. *p < 0.05; ^∗∗^*p* < 0.01; ^∗∗∗^*p* < 0.001.

When creating their goal model, participants in the hierarchical condition focused more on importance, *p* = 0.001; participants in the sequential condition focused more on time, *p* < 0.001. Participants in the network condition did not report focusing more on interconnection relative to the other two conditions, *p* = 0.154. This null difference was likely an artifact due to the interconnection items being relevant not only to the network condition but also to the other two. Specifically, those in the hierarchical and sequential conditions also strongly endorsed the interconnection items (e.g., “I paid a lot of attention to the ways that goals were related to each other.”). Importantly, the results revealed that those in the network condition reported focusing significantly less on both importance, *p* = 0.019, and time, *p* = 0.001. Overall, results suggested that the goal model manipulation successfully induced different focal organizing principles in participants’ goal models.

### Creativity

**FIGURE 6:**
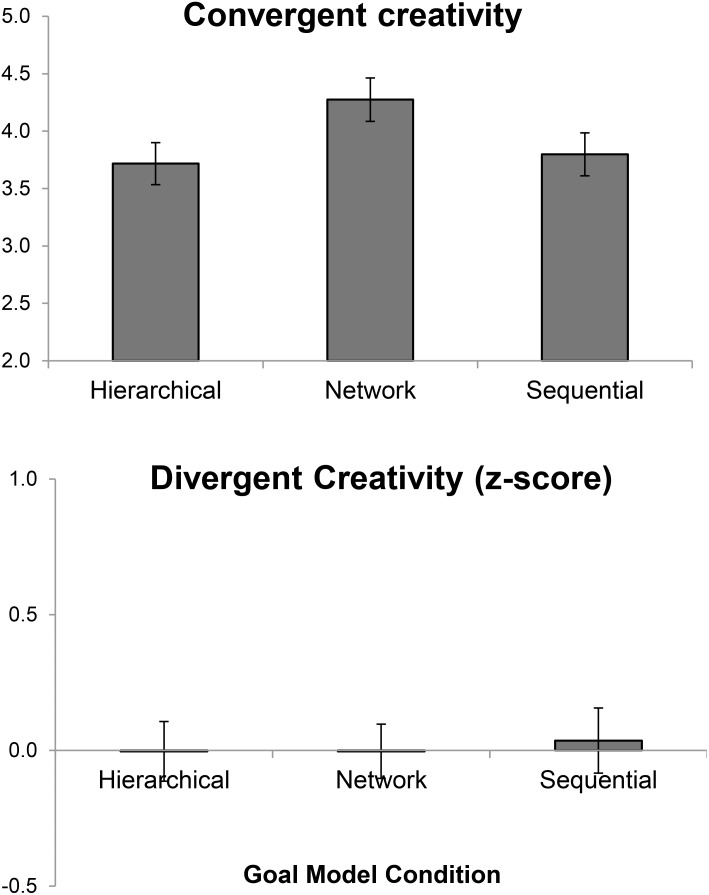
A panel of convergent and divergent creative performance as a function of goal model condition. Error bars = ±1 standard error.

Next, we investigated the creativity outcomes. The zero-order correlation between integrative and divergent creativity scores was *r* = 0.19, *p* = 0.009. This relatively small correlation supported the notion that the two creativity processes are related but distinct from each other (Eysenck, 2003). We hypothesized that a network model might induce greater creative thinking, and particularly so for a convergent creativity task. To test this hypothesis, we conducted *t*-tests using a dummy variable (contrasting network vs. the other two models) to predict the creativity outcomes. Results are illustrated in Figure 6.

Participants in the network condition (vs. the other two) showed a greater performance in the convergent creativity task, *p* = 0.025, $\eta_{p}^{2}$ = 0.03, *d* = 0.34, supporting the hypothesis that a network model can induce convergent creative thinking. In contrast, the network condition did not affect divergent creative performance, *p* = 0.889. This null finding echoed our speculation that there may be a boundary condition for when network models can increase creativity. The full results (including other model comparisons and exploratory analyses) are presented in Table 3.

**Table 3:** Independent *t*-tests and GLMs: network model condition (vs. other two conditions) predicting creativity measures, and exploratory and robustness analyses.

DV	Predictor	*M*	*SD*	*B*	*SE*	*T*	*p*	95% CI	$\eta_{p}^{2}$
**Focal *t*-tests**					
Convergent creativity	(1) Network	4.27	1.41	0.52*	0.23	2.26	0.025	[0.07, 0.97]	0.03
Divergent creativity	(1) Network	-0.003	0.82	-0.02	0.14	-0.14	0.889	[-0.29, 0.25]	<0.01
**Exploratory *t*-tests**					
Convergent creativity	(1) Hierarchical	3.72	1.54	-0.32	0.23	-1.39	0.166	[-0.77, 0.13]	0.01
	(2) Sequential	3.80	1.50	-0.19	0.23	-0.82	0.413	[-0.64, 0.27]	<0.01
Divergent creativity	(1) Hierarchical	-0.003	0.93	-0.02	0.14	-0.15	0.882	[-0.29, 0.25]	<0.01
	(2) Sequential	0.036	0.92	0.04	0.14	0.29	0.772	[-0.23, 0.31]	<0.01
Number of goals	(1) Hierarchical	12.71	5.51	1.66*	0.80	2.08	0.039	[0.08, 3.23]	0.02
	(2) Network	13.45	4.71	2.70***	0.80	3.39	0.001	[1.13, 4.27]	0.06
	(3) Sequential	8.70	4.33	-4.37***	0.75	-5.82	<0.001	[-5.85, -2.89]	0.15
Goal importance	(1) Hierarchical	8.50	1.28	-0.20	0.19	-1.05	0.293	[-0.58, 0.17]	0.01
	(2) Network	8.58	1.01	-0.08	0.19	-0.39	0.695	[-0.46, 0.31]	<0.01
	(3) Sequential	8.82	1.42	0.28	0.19	1.46	0.146	[-0.10, 0.66]	0.01
**Robustness analysis: GLMs including controls^a^**					
Convergent creativity	(1) Network	4.22	1.50	0.45	0.23	1.92	0.057	[-0.01, 0.91]	0.02
	Number of goals			0.02	0.02	0.98	0.326	[-0.02, 0.06]	0.01
	Goal importance			0.18*	0.09	-2.00	0.047	[-0.35, 0.00]	0.02
	(2) Hierarchical	3.66	1.48	-0.41	0.23	-1.82	0.070	[-0.86, 0.03]	0.02
	Number of goals			0.04	0.02	1.72	0.087	[-0.01, 0.08]	0.02
	Goal importance			-0.18*	0.09	-2.02	0.045	[-0.35, 0.00]	0.02
	(3) Sequential	3.91	1.55	-0.01	0.25	-0.03	0.974	[-0.50, 0.48]	<0.01
	Number of goals			0.03	0.02	1.35	0.178	[-0.01, 0.08]	0.01
	Goal importance			-0.17	0.09	-1.93	0.055	[-0.35, 0.00]	0.02
Divergent creativity	(1) Network	-0.07	0.89	-0.11	0.14	-0.76	0.450	[-0.38, 0.17]	<0.01
	Number of goals			0.03*	0.01	2.36	0.019	[0.00, 0.06]	0.03
	Goal importance			-0.07	0.05	-1.30	0.196	[-0.17, 0.04]	0.01
	(2) Hierarchical	-0.05	0.88	-0.08	0.13	-0.61	0.544	[-0.35, 0.18]	<0.01
	Number of goals			0.03*	0.01	2.31	0.022	[0.00, 0.05]	0.03
	Goal importance			-0.07	0.05	-1.34	0.181	[-0.17, 0.03]	0.01
	(3) Sequential	0.15	0.92	0.21	0.15	1.46	0.146	[-0.07, 0.50]	0.01
	Number of goals			0.03**	0.01	2.64	0.009	[0.01, 0.06]	0.04
	Goal importance			-0.07	0.05	-1.35	0.179	[-0.17, 0.03]	0.01

N = 191: 66 hierarchical (35%), 62 network (32%), and 63 sequential (33%). ^*a*^Adjusted means (with control variables) of each goal model condition are reported. Predictors are dummy-coded (e.g., Hierarchical Model: hierarchical = 1, network = 0, sequential = 0). The statistical model(s) tested for each dependent variable are numbered accordingly. *p < 0.05; **p < 0.01; ***p < 0.001.

Robustness analyses were conducted to test the reliability of the observed effect across two potential contingency factors: the number of goals and average goal importance in a goal model. Controlling these factors in the statistical model reduced the significant level of the focal result on convergent creativity (*p* = 0.057) but not the overall pattern of results (Table 3). This suggested that the positive effect of network goal models on convergent creativity performance did not depend on the number of goals participants had and how important the goals were. This result added further support to the argument that it was the properties of participants’ goal model as a whole, rather than properties of their single goals, led to the observed results.

## Discussion

The experiment provided evidence that the use of a network (vs. hierarchical or sequential) model to structure goals increased convergent creative performance. The finding is consistent with the postulation that network models, by placing goals on a relatively equal playing field and highlighting multiple and nonobvious interconnections, boosts the kind of creativity that requires meaningful integration of unrelated ideas. In contrast, the use of a network goal model had no effect on divergent creative performance. Robustness analyses also provided some support for the notion that the effect of network models on convergent creativity is influenced by the overall structure of goals rather than only the independent content of goals.

The study directly contributes to the creativity literature by uncovering a novel antecedent of creativity – goal models. Whereas past creativity research has frequently focused on studying creativity as a dispositional trait (Barron and Harrington, 1981; Runco, 2004b), this research provides an example of how a situational factor can influence creativity. Such impact of situational mindsets on creativity seems to be specific: network goal models increased convergent, but not divergent, creativity. Depending on the nature of the creativity task, different mindsets might be beneficial.

This work generates actionable insights into increasing creativity in organizations. Creativity is a highly desirable ability and predicts extensive benefits for work performance and organizational effectiveness (e.g., good problem-solving, innovations; Anderson et al., 2014). Not surprisingly, many organizations are searching for possible ways to induce creativity at work (Hennessey and Amabile, 2010). The current work suggests that the way in which organizations direct employees to structure their goals (or the ways that employees spontaneously structure their goals) may have downstream implications for creativity. Given a call for creativity-inducing strategies that are both efficient and effective (e.g., Scott et al., 2004), the goal model manipulation may provide a new avenue for boosting (convergent) creativity.

Further, this work extends the organizational literature to study (multiple) goal management. With the increasing usage of diverse visualization strategies to structure goals (Asana, 2018; Mindmeister, 2018b), empirical examinations of the implications of goal models are needed. Our study is one of the first to explore the impact of goal models. It reveals that goal models can affect creativity, exerting unique influence above and beyond the content of people’s goals (controlling for the number of goals and the average importance of goals). Given that organizational research has tended to focus on the study of single goals (e.g., goal setting) or the relation between two goals (e.g., work-family conflict), a focus on goal structure remains an understudied yet potentially influencial topic in this area (Kung and Scholer, 2016). Indeed, our work suggests that the impact of people’s goal structure is likely more than just the sum of its parts.

### Limitations and Future Directions

The current work emphasized internal validity in examining the effect of goal models on creativity. However, as is often the case, there is likely a trade-off between internal and external validity. Regarding goal models, the manipulation task may not wholly reflect the process people typically engage in to structure their goals at work. Compared to existing goal-structuring software (e.g., Asana, 2018; Mindmeister, 2018b), our paper-and-pencil task was relatively easy to administer and intuitive (e.g., no learning of a specific digital interface required), yet it was also potentially less interactive and engaging. More research is needed to examine whether these differences matter and to what extent the network model effect on creativity is generalized in the use of a computerized goal-structuring software. Regarding creativity, the thinking processes involved to perform well in these standardized tasks should be theoretically transferable to solving spontaneous creative demands at work. Yet we do not know for sure whether these tasks adequately represent creative challenges in the real world. Future research using a more ecologically valid measure will help address these limitations, including the assessment of employees’ creativity in their business development plans, product designs, or team innovations.

In addition, there is still much to learn about the mechanisms underlying the goal model effect on creativity. Future work should investigate the psychological mechanisms through which network models affect creativity (e.g., weaker bias against connecting unrelated ideas, recognizing multiple and nonobvious interconnections). Additionally, two other interesting effects emerged from the exploratory analyses. First, goal importance appeared to have an independent negative effect on convergent creativity – the more a person’s goals were important (on average), the worse their convergent creativity performance. Second, the number of goals in a goal model seemed to affect divergent creativity positively – the more goals a person had, the higher their divergent creativity performance. This association might be a result of people who were more generative in terms of thinking of both goals and ideas spontaneously. These could be exciting directions to explore.

Furthermore, more research is needed to understand the utility and limits of the use of network models. Although our study uncovered one boundary condition of the network model effect on creativity – the kind of creativity involved – other moderators have yet been explored. Searching for moderators has important practical implications, so that one can maximize the benefit (and minimize the potential drawbacks) of the use of network models. For instance, although there is evidence that network models facilitated convergent creative thinking, the effect size was small-to-medium ($\eta_{p}^{2}$ = 0.03 or *d* = 0.34), and the current study does not test how long the manipulation would affect subsequent behavior. To address these issues, future research should test the duration of the manipulation effect, as well as investigate ways to increase the effectiveness of the manipulation.

In addition, as noted earlier, although there is evidence that goal models can be distinct entities (Kung and Scholer, 2016), this does not preclude the possibility that people form integrated goal models (i.e., using multiple goal organizing structures simultaneously; e.g., Nevogt, 2015). Integrated models may have their own distinct effects. For instance, a hierarchical-network model could show an additive effect of the implications of each model individually, or a hierarchical-network model could show interactive effects, such that new implications are revealed. Future work will benefit from a deeper understanding of these nuanced goal model effects.

Finally, our experiment is limited by the size and diversity of the sample. A replication with a larger sample would be ideal to detect the goal model effect on creativity more reliably. With the observed effect size, *d* = 0.34, this would mean about 268 people for 80% power at the 0.05 alpha error probability (two-tailed; Faul et al., 2009). Although our sample was culturally diverse (e.g., 35% White, 48% Asian), most participants were female (72%) and all were undergraduate students in Canada. Therefore, future work should explore whether the observed effect would generalize to other populations (e.g., people in different countries, of different ages, or in different occupations).

## Conclusion

Employee creativity offers excellent benefits to organizations, and the current work adds new insight into how creativity can be facilitated through the way people structure their goals. Goal model induction (and increasingly popular goal-structuring software) presents a potential avenue for organizations to unlock workers’ creative potential. By increasing people’s awareness of the connections between seemingly unrelated ideas via network goal models, greater organizational effectiveness may be achieved.

## Ethics Statement

This study was carried out in accordance with the recommendations of the Human Research Ethics Committee at the University of Waterloo with written informed consent from all subjects. All subjects gave written informed consent in accordance with the Declaration of Helsinki. The protocol was approved by the Human Research Ethics Committee at the University of Waterloo.

## Author Contributions

Both authors conceptualized the idea and collected the data. FK analyzed the data and drafted the manuscript. AS provided critical feedback on the manuscript.

## Conflict of Interest Statement

The authors declare that the research was conducted in the absence of any commercial or financial relationships that could be construed as a potential conflict of interest.

## Footnotes

1 https://www.mindmeister.com/

2 http://www.airtable.com

3 https://asana.com

4 https://www.targetprocess.com

5 https://trello.com

6 Subsequently, unrelated to the manipulation, participants also were instructed to use different color pens to make indications on their goal map for additional information about the goal relations. Critically, participants were told not to add or erase any goals already on their goal model, and only make indications of the goal relations. Specifically, they used a blue pen to indicate the valence of any existing relations among their goals and a red pen to indicate any new relations. They were told to use a “+” sign to indicate cases where the pursuit of a given goal facilitates/helps the pursuit of the associated goal; to use a “-” sign to indicate cases where the pursuit of a given goal hinders/excludes the pursuit of the associated goal.

7 Prior to the study, we conducted scale validation analyses. In short, using a separate sample (*n* = 495), we conducted an exploratory factor analysis and observed that the three goal model subscales emerged to be three unique factors (average eigenvalues = 2.98). Further, in another sample (*n* = 515), confirmatory factor analysis results demonstrated that the subscales were related to but distinct from each other, and the current configuration produced the greatest model fit (CFI = 0.989, PCLOSE = 0.947, RMSEA = 0.037).

## Appendix A

### Goal Model Scale

1. My goals were categorized from the most important to least clearly.
2. There was a clear hierarchy of goals. Some goals were just meant to be more important than the others.
3. I classified my goals by order of importance.
4. I organized my goals by significance from the most important to the least.
5. I organized my goals based on their level of interconnectedness.
6. I paid a lot of attention to the ways that goals were related to each other.
7. Many of my goals were closely related to others, and therefore completing a goal could greatly influence the progress towards its connected goals.
8. The more important my goal was, the more it would be interconnected with and influence other goals.
9. I placed my goals on a timeline based on the chronological order to achieve them.
10. My goals were arranged in a step-by-step process.
11. I organized my goals in chronological orders.
12. My drawing of the goals had a clear sequence of steps.

Used as a manipulation check. The hierarchical model sub-scale consists of Item 1–4; the network model sub-scale consists of Item 5–8; the sequential model sub-scale consistent of Item 9–12.
