# Supplementary material for: A Network Model of Goals Boosts Convergent Creativity Performance
Source: Front Psychol. 2018 Oct 29;9:1910. doi: 10.3389/fpsyg.2018.01910 (PMC6232898; doi:10.3389/fpsyg.2018.01910)
Supplement: Supplementary file 1 [file Image_1.pdf]

Supplementary Materials

A Network Model of Goals Boosts Convergent Creativity Performance

Franki Y. H. Kung   Abigail A. Scholer

Contents

Goal Model Manipulation 2
A Sample Goal model from Each Goal Model Condition 5
Story Rewriting Task (Convergent creativity) 6
Brick Use Task (Divergent Creativity) 8

Goal Model Manipulation

The Network Model Condition

In this task, we want to understand how students organize what they do at school to achieve their goal of academic success. In the space below, please think about your goal of academic success, and produce a diagram that visually represents what you usually do or you can do to achieve it.

Read the information and follow the structure of the figure to create your diagram.

Organize Your Goals as a Network

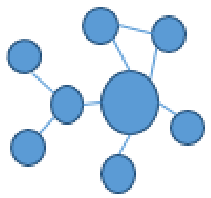

Provide a clear clustering structure, which means goals are related to or derived from each other. Goals have different degrees of connections, and goals that are more closely related are tied and together form clusters. For example, to achieve good health, people could eat healthy food and rest well every day (associated goals).

There are no right or wrong presentations. Try your best to be comprehensive and create as many goals and links associated with academic success as you want. Please label all elements of your diagram.

Your representation of achieving academic success:

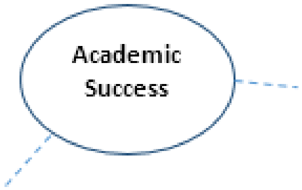

The Hierarchical Model Condition

In this task, we want to understand how students organize what they do at school to achieve their goal of university success. In the space below, please think about your goal of achieving success in university (e.g., academic, health, social etc.), and produce a diagram that visually represents what you usually do or you can do to achieve it.

Read the information and follow the structure of the figure to create your diagram.

Organize Your Goals on a Hierarchy

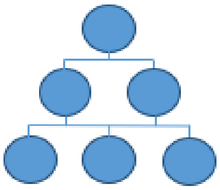

Provide a clear ordering structure, which means the higher-order goals are superior to and encompass the lower-order ones. Lower-order goals ae the means to the higher-order ones. For example, to achieve good health (as a higher-order goal), people could eat healthy food and rest well every day (as lower-order goals).

There are no right or wrong presentations. Try your best to <u>be comprehensive and create as many subgoals under academic success as you want</u>. Please <u>label all elements</u> of your diagram.

Your representation of achieving university success:

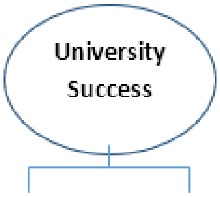

The Sequential Model Condition

In this task, we want to understand how students organize what they do at school to achieve their goal of university success. In the space below, please think about your goal of achieving success in university (e.g., academic, health, social etc.), and produce a diagram that visually represents what you usually do or you can do to achieve it.

Read the information and follow the structure of the figure to create your diagram.

Organize Your Goals in a Sequence

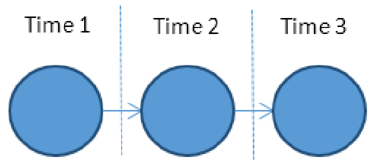

Provide a clear sequential structure, which means goals are organized along the timeline or in stages. The attainment of one goal depends on the attainment of the prior goal. For example, to achieve good health (the end-goal), people take different prior steps - eat healthy food and/or rest well every day.

There are no right or wrong presentations. Try your best to be comprehensive and create as many steps that lead to your success in university as you want. Please label all elements of your diagram.

Your representation of achieving university success:

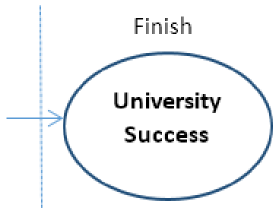

A Sample Goal model from Each Goal Model Condition

<table>
<tr><td>Hierarchical</td><td>
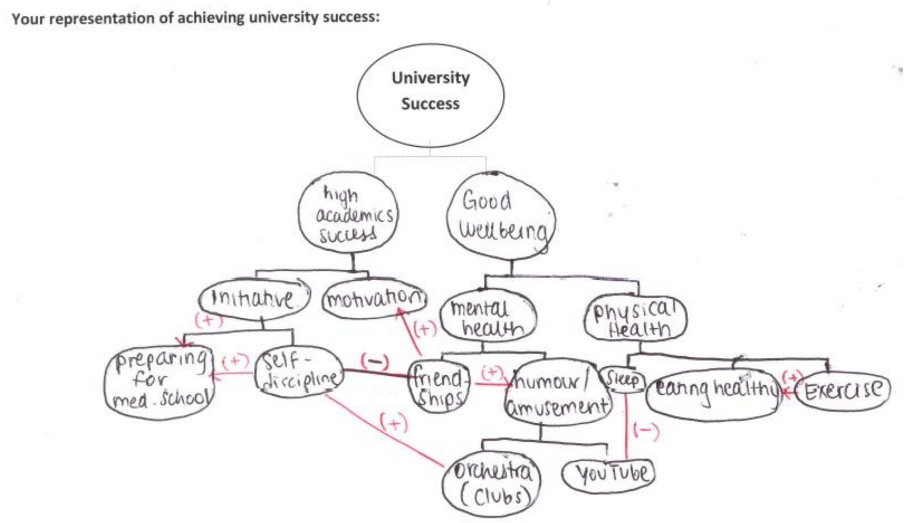

</td></tr>
<tr><td>Network</td><td>
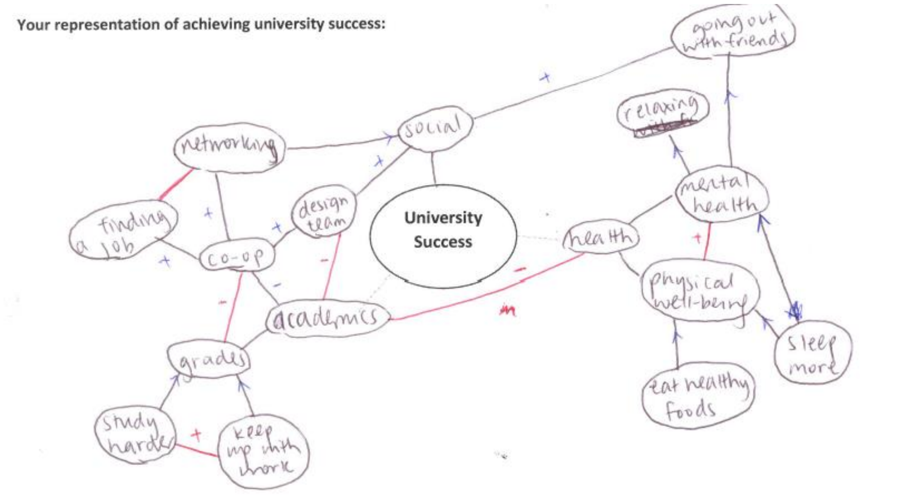

</td></tr>
<tr><td>Sequential</td><td>
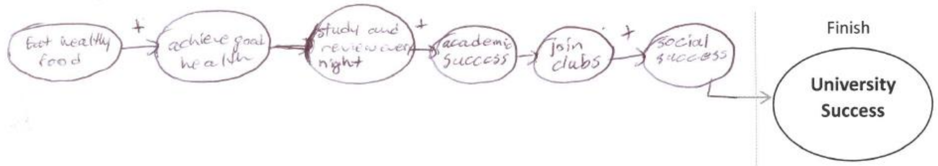

</td></tr>
</table>

Story Rewriting Task (Convergent creativity)

Creative Story Rewriting

In this task, we want to study people's imagination.

You will read a summary of a fairy tale randomly selected by the computer. After reading, spend a few minutes on thinking about how *you* would develop a new version of it. You may use your wildest imagination to rewrite the story, and the story should be developed from the original fairy tale.

Try your best to be creative, original, and coherent! Please do not worry too much about grammar as long as you think the ideas are clear.

>>

The Story of Snow White

Once upon a time, there was a queen who named her only daughter Snow White because of her beautiful skin. The queen died, and Snow White's father married a new queen, who was evil and vain. Every morning she would stand in front of the mirror and say, "Mirror, mirror on the wall, who is the fairest one of all?" The mirror always answered, "you are," until one day it said that Snow White was the fairest one of all.

The evil queen ordered one of her servants to take Snow White into the forest to have her killed. The servant, feeling sorry for Snow White, let her go and brought back a boar's heart to show the queen he had done the deed. Snow White, alone and hungry in the forest, came across a little cottage with seven dwarves. They said she could stay with them if she cleaned and cooked.

They all lived happily until one day when the mirror told the evil queen that Snow White was still alive. The evil queen disguised herself as an old lady, went to the cottage, and gave Snow White a red apple that was poisoned.

When Snow White took a bite of the apple, she fell down unconscious. The dwarves were very sad and built a glass coffin for her. One day a prince came by and saw how beautiful Snow White was, and bent down to give her a kiss. Snow White woke up, and they were married.

Your version:

Once upon a time....

Brick Use Task (Divergent Creativity)

The following is a timed task. You are given 2 minutes in the task. Please read the below information carefully before you start.

You will be randomly presented with an object. Please try your best to list as many creative uses of that object as possible. Note that the uses of the object you give are not limited to any kinds or to any uses you had seen or heard about before.

When you are ready, please click to start. Once you have clicked the next button, you will know what is the object and the timer will start counting down.

>>

0153

Use of a Brick

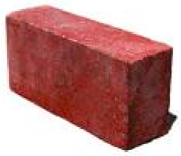

Think of a brick. What are the different ways in which you could use a brick?
Please write down **all the uses that come to your mind**. Please begin now.

[The ask will end automatically after 2 minutes.]

1	
2	
3	
4	
5	
6	
7	
8	
